# A scoping review to characterize bridging tasks in the literature on aging with disability

**DOI:** 10.1186/s12913-020-5046-5

**Published:** 2020-03-04

**Authors:** Emily Joan Nalder, Tyler M. Saumur, Zinnia Batliwalla, Luis Salvador-Carulla, Michelle Putnam, Andria Spindel, Erica Lenton, Hamdi Hussein

**Affiliations:** 1 Department of Occupational Science and Occupational Therapy, University of Toronto, 160-500 University Ave, Toronto, ON M5G1V7 Canada; 2 Rehabilitation Sciences Institute, University of Toronto, 500 University Ave, Toronto, ON M5G1V7 Canada; 3 March of Dimes Canada, 10 Overlea Blvd, East York, ON M4H 1A4 Canada; 4 Centre for Mental Health Research, Research School of Population Health, College of Health and Medicine, The Australian National University, 63 Eggleston Rd, Acton, Canberra, ACT 2601 Australia; 5 School of Social Work, Simmons College, 300 The Fenway, Boston, MA 02115 USA; 6 Gerstein Science Information Centre, University of Toronto, 9 King’s College Cir, Toronto, ON M7A 1A5 Canada

**Keywords:** Bridging, Aging, Disability, Collaboration, Knowledge exchange

## Abstract

**Background:**

Bridging involves improving knowledge sharing and collaboration across different fields, such as aging and disability. The objectives of this review were to describe: 1) the contexts where bridging has occurred in relation to delivery of health services for adults aging with neurological or developmental conditions; and 2) characterize and map bridging tasks, stakeholders involved, and outcomes discussed in peer-reviewed literature.

**Methods:**

Seven databases were searched around the core concepts of “bridging,” “aging,” and “disability.” In total, 10,819 articles were screened with 49 meeting the inclusion criteria of discussing aging with developmental or neurological disability, explicitly describing bridging tasks, published in English and a peer-reviewed publication. Bibliographic information, sample characteristics, and data on bridging was extracted and included in the qualitative synthesis.

**Results:**

Intellectual and/or Developmental disabilities were the most studied population (76% of articles), and most articles were published in the United States (57%). Twenty-two bridging tasks were identified, and categorized into three domains: health and social service delivery (e.g., care coordination tasks), policy (e.g., policy change), and research and training (e.g., mentoring). Stakeholders involved ranged from health care professionals to policy makers and organizations in aging and disability services.

**Conclusions:**

The resulting matrix will assist in the specification of bridging in research and practice. Future work should evaluate specific models of bridging and their effects on health service delivery.

## Background

Bridging as a field of scientific knowledge considers how silos can be broken down, and how individuals from different fields can work together sharing their knowledge and skills to address complex issues. ‘Aging with disability’ refers to the study of the processes and experiences of aging, in the context of long-term disability, and this notion has driven much of the interest in the construct of bridging in the health care setting [1, 2]. In this article the concept of bridging is applied to the aging and disability fields [3].

Bridging emerged in the context of increasingly complex health systems that need to adapt to rising demands and changing consumer needs. Aging populations place an added demand on health services, as rates of disability and health care utilization are high in older populations [2, 4, 5]. Simultaneously life expectancies have increased, meaning individuals living with disability acquired earlier in life may require services and supports to maintain their health and wellbeing for a longer period [1, 6]. In addition, their health care needs can become more complex over time due to secondary complications or the onset of other health conditions associated with aging [1, 5]. While the converging demands of aging and disability for health systems are apparent, many authors note that the aging and disability systems have historically developed in parallel, following different pathways [5]. There have been calls for bridging of aging and disability networks in order to learn together, develop a shared agenda and potentially realize opportunities for policy and program development meeting the needs of individuals aging with disability [5].

The literature on aging with a disability has called for bridging activities to address separated knowledge bases, service systems, and policies, for individuals living with disability and older adults [7], and there are examples of policy changes, particularly in the US, spurring bridging of aging and disability networks. In 1999, the Olmstead decision of the US Supreme Court supported the rights of individuals of all ability levels to live in the community. Bridging in this scenario was the policy change, where age- and disability-related groupings were disassembled to create policies reflecting needs of all individuals [7]. Additionally, the formation of the Administration on Community Living in the United States represented a clear example of changing organizational structures that may contribute to the degradation of silos separating aging and disability services [7]. This led to other bridging initiatives in the US, including the expansion of shared resources through centers such Aging and Disability Resources Centers [8]. Operations such as these can provide appropriate access to services and supports that is vital for older adults and those with disability. Given the discussion of bridging activities in the literature, and different examples of bridging in practice, it is pertinent to synthesize the evidence and characterize specific examples of bridging so they can be scrutinized in terms of how does bridging occur (through what tasks or activities), and with what effect.

An interdisciplinary group of twelve experts in aging and disability engaged in a year-long, iterative process to define bridging through discussions among organizers and attendees of a conference, ‘Growing Older with a Disability (GOWD), Festival of International Conferences on Caregiving, Disability, Aging and Technology (FICCDAT)’ held in 2011. The result was a published declaration – the Toronto Declaration – which defined bridging as ‘a range of concepts, tasks, technologies, and practices aimed at improving knowledge sharing and collaboration across stakeholders, organizations and fields in the care and support of persons with disabilities, their families and the aging population’ ([4], p., 2). Bridging as an approach seeks common ground, recognizing that the aging and disability fields have overlapping and intersecting issues that can be addressed through the utilization of knowledge from both fields [3, 4]. The Toronto Declaration identified five priority areas for bridging (see Table 1) [3]. The multiple and complex challenges facing individuals aging with disability is reflected here, as bridging can address a range of needs (e.g., bridging may be required to improve access to long-term care and supports, ensure financial security, or maintain health and wellbeing through health promotion or early identification and management of secondary conditions). Different tasks may be required to address each of these identified priority needs including policy development and analysis, knowledge transfer and dissemination, care coordination, and financing of health and social services to incentivize collaboration [4]. The authors noted that the range of possible bridging tasks are vast and that there is an urgent need to better catalogue these tasks for the evaluation, and broad implementation of successful bridging models in the aging and disability context [3]. The bridging tasks identified in the Toronto Declaration emerged through discussions with experts in the field and are therefore based on their knowledge of evidence and bridging in practice.

**Table 1:** Priority areas for bridging as defined in the Toronto Declaration (2012)

Bridging Target	Definition
Health and well-being	Improved access to health care services; improved diagnosis and treatment of secondary conditions and diseases; care coordination; health literacy; health promotion and wellness; prevention of age-related chronic conditions; prevention of abuse and neglect; reduction in pre-mature mortality and training of health professionals in aging and disability.
Inclusion, participation and community	Accessible societies, including age and disability friendly communities, removal of barriers of any kind: architectural, cultural, legislative. Impact and implications of aging and disability on civic and community engagement, and the role of technology and universal design in fostering inclusion, participation and knowledge management.
Long-term supports and services	Support for families and caregivers, training and education of direct support professionals; self-determination, access, availability, and affordability of supports and services; ethical issue related to non-discrimination, such as in palliative care, end of life issues.
Income security	Employment, retirement security, asset development; accommodation and accessibility in the work setting; value of non-paid social and community contributions.
Science of bridging	Research on bridging aging and disability and on ways to transfer this knowledge locally, nationally, and internationally to policy development.

A next step is to characterize and classify these bridging tasks drawing on published evidence, framing the scientific knowledge pertaining to bridging. Framing scientific knowledge is a specific type of research which aims to outline and organize related knowledge bases [9]. Framing studies contribute towards the development of research questions to build an understanding of complex phenomena. Examples of framing studies include consensus statements and studies to map concepts and develop classification schemes (such as a taxonomy or typology) [9].

In summary, bridging has been called for and enacted in policy and practice, to break down aging and disability silos and promote equitable access to services and supports, based on the recognition that community participation for *all* is the common goal irrespective of age or disability labels. The purpose of this scoping review was to synthesize the literature on aging with disability, and characterize bridging tasks and research gaps that require further study. Specific objectives of the review were to describe: 1) the contexts where bridging has occurred (e.g., year and country of publication, populations studied, priority areas discussed); and 2) the bridging tasks, the stakeholders involved, and the intended outcomes of bridging discussed in peer-reviewed literature. This work would delineate key concepts describing bridging tasks so that they can be investigated in future research.

## Methods

### Protocol

The protocol for the present scoping review was established using the guidelines proposed by Arskey and O’Malley [10] and updated by Levac and colleagues [11]. The establishment of the protocol involved specifying our purpose and research question, developing a search strategy, selecting appropriate and relevant works, extracting data, and summarizing and reporting the results. A full version of this protocol has been published previously [12].

### Search Strategy & Search Terms

The following databases were searched for peer-reviewed journal articles focused on bridging aging and disability: Ovid Medline Epub Ahead of Print, In-Process and Other Non-Indexed Citations, Ovid Medline Daily and Ovid Medline (1946 to present), Ovid Embase (1947 to present), Ovid Allied and Complementary Medicine (AMED) (1985 to present), Ovid PsycINFO (1806 to present), EBSCO Cumulative Index to Nursing and Allied Health Literature (CINAHL; 1981 to present), ProQuest Sociological Abstracts (1952 to present), and the Cochrane Library. The publication period was from inception until March 2017. Search terms were developed by a research librarian (EL) with expertise in rehabilitation science. Search terms were constructed based upon the core concepts of bridging, aging, and disability. All searches were conducted by a member of the research team (ZB). An example of a search strategy can be found in a previously published protocol [12].

### Study selection & screening

The following inclusion criteria were used to identify relevant articles: [1] Article was published in English, [2] Article addressed aging with a neurological or developmental disability, [3] Article explicitly discussed bridging tasks such as purposeful knowledge exchange, partnership development, or collaboration activities, [4] Article used qualitative, quantitative, mixed-methods designs, or was an editorial or commentary. Grey literature (e.g., white papers, government publications) were excluded as the purpose of the review was to discuss bridging tasks described in peer-reviewed literature. Due to the high volume of literature related to disability, the inclusion criteria operationalized ‘aging with disability’ in terms of populations with developmental or neurological conditions. Using this criteria, articles discussing disability in relation to chronic disease, musculoskeletal conditions, sensory loss or mental health conditions were excluded. The decision to focus on literature pertaining to developmental or neurological conditions was because these populations were known to have studies discussing issues related to aging with disability.

Records identified in the database searching were uploaded into EndNote [13] and were de-duplicated. Titles and abstracts were then uploaded into Covidence systematic review software, for screening. A two-step process was used to screen articles. First, four members of the research team independently screened the articles titles and abstracts (HH, ZB, EN, TS), with at least two team members reviewing each article and then comparing their decisions to include or exclude. If there was disagreement between the two reviewers, an additional team member was consulted to reach consensus on the inclusion or exclusion of the article.

Following title and abstract screening, the resulting articles were distributed amongst the reviewers in pairs for independent review of the full text. Discrepancies were discussed to reach a decision and a third reviewer provided input if the pair failed to reach consensus. If articles were not selected, a reason was provided based on the inclusion/exclusion criteria. Once articles were included following full-text screening, the Scopus database was searched to include any articles which had cited the included articles, or that the included articles had referenced. Subsequent title/abstract screening and full-text screening was performed on these articles using the methods outlined earlier.

### Data extraction

A data extraction form was developed using an iterative process whereby two reviewers (ZB, EN) independently extracted data from a random sample of 10 articles. Once the data extraction sheet had been developed, piloted, and the two reviewers were in agreement regarding what data was extracted from each article, the form was implemented for all included articles. The following items were extracted for data analysis: [1] Bibliographic information, [2] Methodological information, [3] Population studied, [4] Context of bridging, [5] Bridging tasks discussed. Similar to full-text screening, the included articles were split amongst pairs of reviewers (HH, ZB, EN, TS) for independent extraction of the relevant information. Discrepancies were discussed amongst the pair and if unable to reach consensus, an additional reviewer was consulted to reach agreement.

### Data analysis

**Fig. 1:**
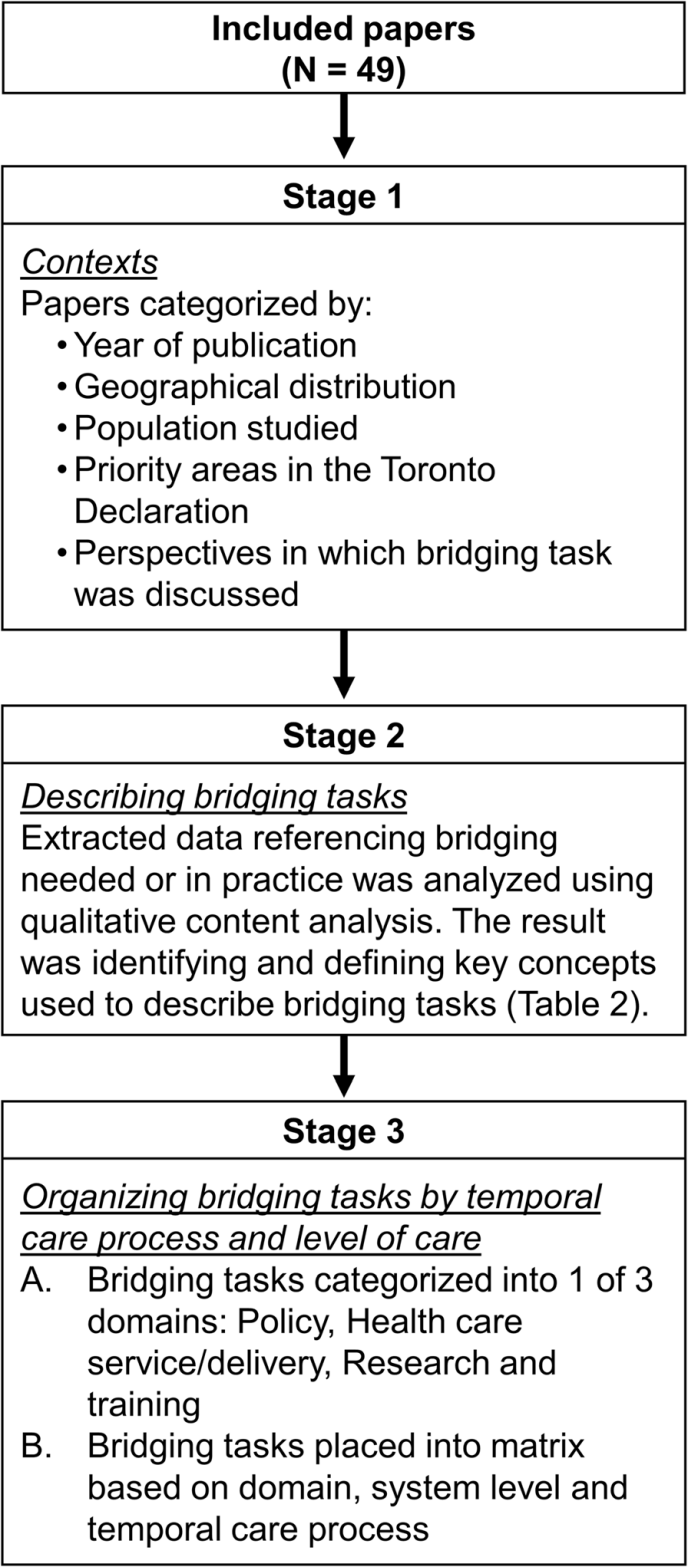
A schematic outlining the different stages of data analysis

Data analysis involved three stages as reflected in Fig. 1. Stage 1 was a descriptive stage of analysis, to provide an overview of the state of the literature describing bridging tasks and the contexts where the tasks occurred (stage 1). This was done to identify areas of strength and gaps in knowledge. Stages 2 and 3 involved an iterative process of qualitative analysis to describe the bridging tasks emerging from the literature (stage 2), and to separate the tasks into domains and organize the tasks in a matrix to show how the tasks were being utilized (i.e., at different geographic levels, and how they unfolded temporally) (stage 3). Stage 2 was therefore contributing to a clear language for describing bridging tasks and could be used to compare with the tasks discussed in the Toronto Declaration. Conversely, stage 3 contributes to a more nuanced understanding of how these tasks were used including, the different levels of care covered by the bridging initiatives (or not), and the temporal aspects thereby recognizing that these tasks occur as a process involving varied inputs and leading to different outputs/outcomes. Each stage is described in more detail below.

To address objective 1 and describe the contexts where bridging has occurred, a descriptive analysis was performed (stage 1) whereby studies were classified based upon a number of elements such as the country where research was conducted, the decade in which the research was published, the population being studied, the priority areas for bridging based on definitions outlined in the Toronto Declaration [3](see Table 1), and whether the literature described: bridging tasks needed (calling for more resources and efforts to implement them), initiatives occurring in practice (promising practices or examples of bridging on the ground), or the evaluation of bridging tasks in the article.

To address objective 2 and describe the bridging tasks, stakeholders involved, and intended outcomes, a qualitative content analysis was used (stage 2) [14]. Extracted data was read several times to increase familiarity with the information. Data on the study information and bridging activities that were extracted were then uploaded into NVivo (NVivo-10, QSR International, Melbourne, Australia). Initial coding was used to develop an initial list of concepts and terms describing the bridging tasks. These concepts were initially grounded in the data and not based on any prior frameworks (e.g., the Toronto Declaration). The concepts were discussed with team members, and then organized, grouping similar concepts together into higher and lower order categories. Once the list of concepts had been refined and organized into categories, the ideas were compared to other frameworks and definitions for the concepts were developed based on terminology taken from the International Classification of Health Interventions [15, 16] and a case management taxonomy [17]. The Toronto Declaration [3] was referred to at this stage for reflection only, as whilst they defined priority areas for bridging (see Table 1), no definitions for specific bridging tasks (e.g., dissemination or coordination) were provided in that paper. Any new concepts emerging from the data were defined by team members. Coding was iterative and once clear definitions of each concept were developed, three team members (EN, ZB, TS) went back to the extracted data applying the concepts developed to confirm they accurately reflected all of the bridging tasks described in the included articles. Regular meetings among all team members allowed for discussion and refinement of the concepts and their definitions, ensuring that each concept was distinct, and to theorize relationships between the concepts when relevant.

Once the key concepts reflecting bridging tasks were finalized in stage 2, the team organized coded data by applying ecological production theory [18] to health and social care using the matrix developed by Tansella and Thornicroft (stage 3) [19]. This matrix reflects both the temporal care processes (input, actions or throughput, and output/outcome), and ecosystem/geographic levels of care (macro or country; meso or community; micro or team/organization; and nano or patient level) [20] In the present study, the matrix was adapted to describe bridging activities according to different domains (research and education, service delivery and policy), and allowed us to reflect the different tasks as they occurred in each domain, at different levels of care, and through different temporal processes.

## Results

**Fig. 2:**
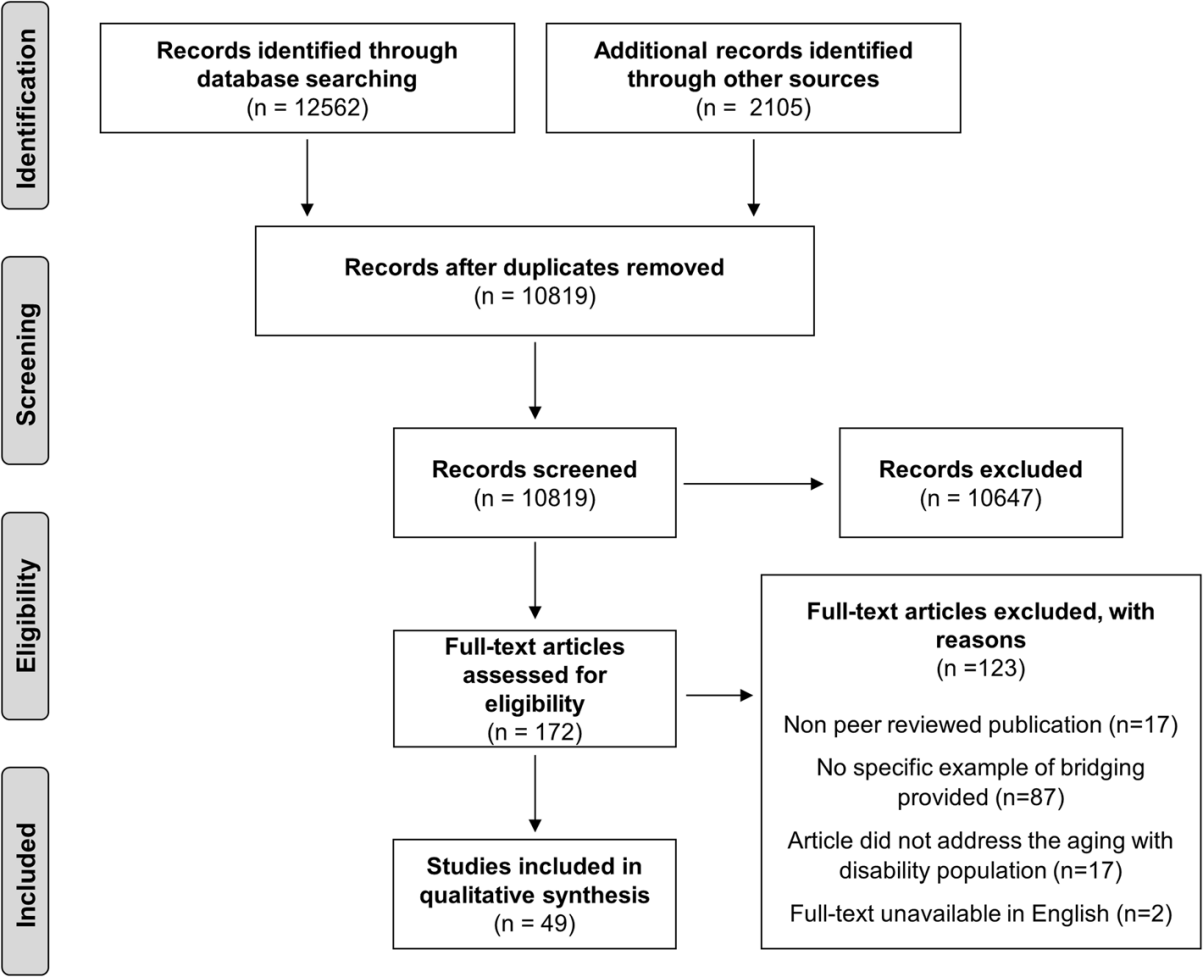
PRISMA Diagram

The implemented search resulted in an initial 12,562 articles being identified with 49 ultimately being included for analysis [3, 7, 8, 21–65]. Fig. 2 outlines the reasons articles were excluded. Commentaries, policy analysis, discussion or theoretical papers were common (*n* = 25, 51%). Other articles (*n* = 22, 45%) presented original research, including qualitative methods (*n* = 8), case studies (*n* = 7), mixed or multiple methods studies (*n* = 4) or quantitative methods (*n* = 3). The remainder of the included papers were review papers or knowledge syntheses (*n* = 2, 4%). Details of all included articles, and a description of the bridging tasks described, are provided in Additional file 1: Appendix A.

### Contexts of bridging

#### Year of publication and geographical distribution

**Fig. 3:**
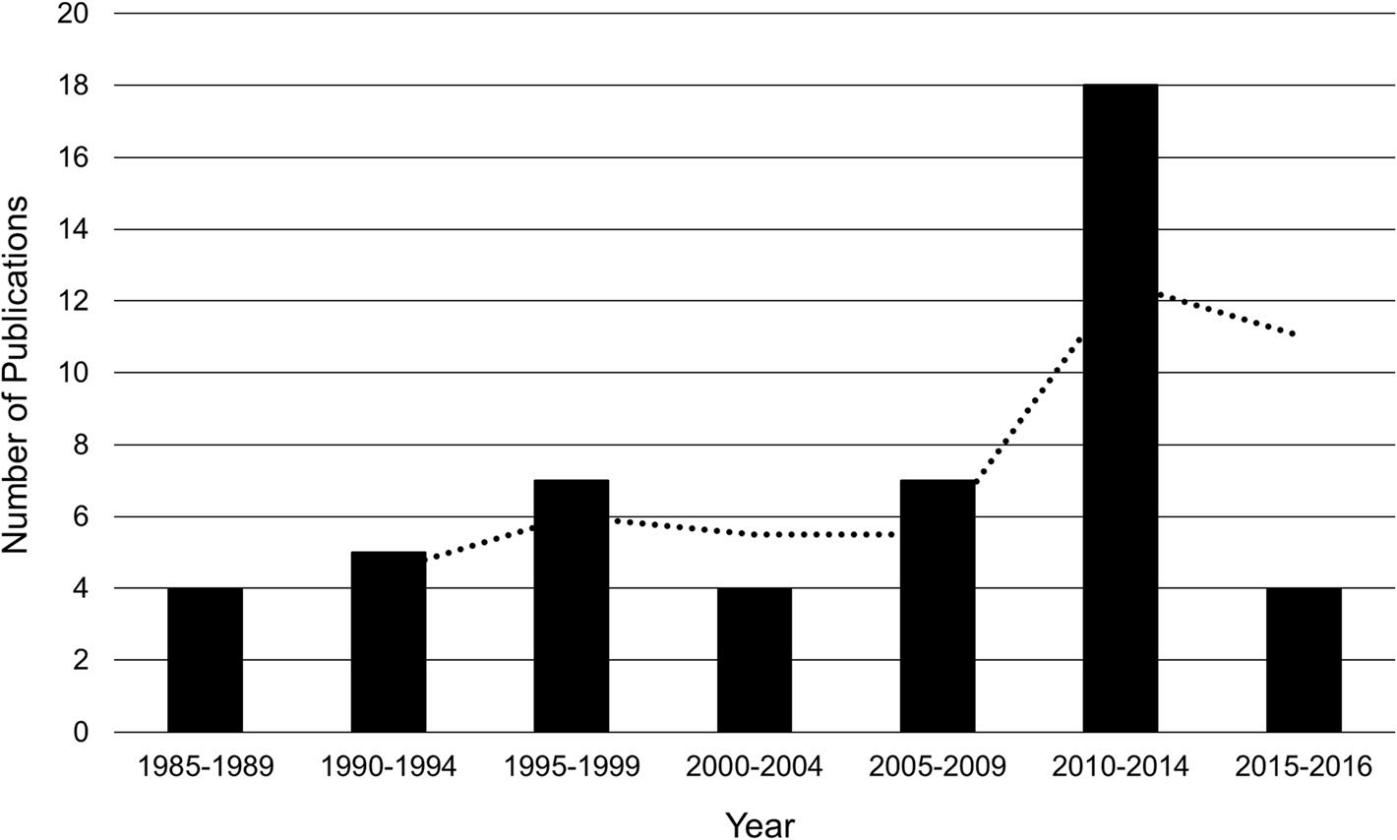
Number of articles published per five-year period

Fig. 3 depicts the proportion of included studies published in 5-year periods. The majority of studies were published after 1999 (*n* = 33, 67%), whereas only 4 (8%) of the included papers were published before the year 1990. The majority of the described articles were produced in the United States (*n* = 28, 57%), with a quarter being produced in Australia (*n* = 12, 25%). The remaining articles were composed of work produced in in Europe (*n* = 8, 16%): Ireland (*n* = 5, 10%), the UK (*n* = 2, 4%), Spain (*n* = 1, 2%); and Canada (*n* = 1, 2%).

#### Populations studied

The majority of research described the population as either individuals with Intellectual Disability (*n* = 12, 24%), Developmental Disability (*n* = 11, 22%), or individuals with Intellectual and/or Developmental Disability ([I/DD]; *n* = 10, 20%). An additional 5 studies (10%) included individuals with both Intellectual Disability and Dementia. The remaining studies specified the population as: individuals ‘aging with disability’ (used to describe individuals in broad terms such as, individuals living with physical or cognitive disability, without specifying specific disorders; *n* = 7, 14%); individuals with ‘lifelong disability’ (a range of conditions, such as brain injury or cerebral palsy, that result in functional impairments that persist over the lifespan; *n* = 3, 6%); or neurological conditions (a range of neurological conditions such as brain injury, Multiple Sclerosis or Spinal Cord Injury; *n* = 1, 2%). In the I/DD literature, the rationale for bridging aging and disability was strongly driven by the improvement in health care for those living with I/DD, leading to this population living longer.

#### Priority areas for bridging

Twenty-three of the included studies (47%) discussed bridging activities in the area of long-term supports and services. For example, bridging tasks were proposed to address supports for families and caregivers, the quality of training of direct support professionals, and access to long-term supports, including palliative and end-of-life care for those aging with disability. Fifteen studies (31%) discussed bridging activities in the area of health and well-being. These included bridging tasks that were purported to address early diagnosis and management of chronic or age-related conditions, and health promotion for individuals aging with disability. Eleven studies (22%) described bridging in the area of inclusion, participation, and community living. For example, bridging could target access to employment, retirement and/or housing services for individuals with disability, and the accessibility of societies. No studies had a primary focus on bridging to address income security or the science of bridging.

#### Perspectives of bridging tasks discussed

Bridging tasks were discussed in three different perspectives in the included articles: bridging tasks currently in practice (*n* = 34, 69%); bridging tasks evaluated (*n* = 21, 43%); or bridging tasks called for or viewed as needed (*n* = 19, 40%). Examples of bridging tasks that were being discussed currently in practice were mandated joint planning initiatives between sectors [24], specific personnel such as registered nurses in ID who have the capacity to bridge aging and ID [36], and funding for cross-disability research and training centers on aging with a disability [64]. Bridging tasks reviewed in original research studies included pilot projects involving joint programming between disability services client and aged care residents [34, 47], a palliative care educational program for staff caring for those with Intellectual Disabilities [37], and area agencies incorporating linkages with Developmental Disability agencies [46]. Examples of bridging tasks that were being called for were policy development and funding mechanisms covering aging and disability housing services, to enable aging in place for those with I/DD [26], training across staff regarding physical, cognitive and social changes in older adults with disabilities [8, 39] and collecting data from individuals aging with disability to determine unmet needs and identify best practices [7]. Twenty-three (47%) of the included studies discussed bridging tasks in more than one of these perspectives, as either the article reported on multiple bridging tasks, described pilot initiatives or promising examples of bridging in practice but also called for additional attention and resources for bridging [8, 26, 48, 49], or referenced examples of bridging in other countries/jurisdictions but called for more work to bridge aging and disability in their setting [50, 52, 57]. There were no apparent trends over time, as the median year of publication for articles evaluating bridging tasks, and those calling for bridging to occur was 2008.

### Bridging tasks described in the literature

Table 2 presents definitions for the twenty-two main concepts used to describe bridging tasks that emerged through the analysis, and therefore reflect examples of bridging discussed in the literature on aging with disability. In total, 445 mentions of these bridging tasks were in the included articles. The tasks most commonly described were partnerships and collaboration (*n* = 82 occasions; 18%), training and skills development (*n* = 39 occasions; 9%) and facilitation (*n* = 37 occasions; 8%). Nine of the bridging tasks reflect actions linked to case management, including referral, assessment, navigation, facilitation, advocacy, advising, planning, collaboration, and training. Two bridging tasks, adapting services or systems to make them accessible to individuals with disability and establishing health (related) services were not present in other frameworks describing health interventions. These concepts are broader categories involving a number of sub-tasks (e.g., planning, collaboration) involved in modifying services or systems for older people so they are accessible to individuals with disability, or to create new programs to meet unique needs of those individuals aging with disability. Pre-requisites, or inputs, necessary for bridging were also identified, such as knowledge, and a supportive organizational culture (see Table 2).

**Table 2:** Key concepts related to bridging aging and disability

	Concept (# of occasions /445)	Definition
Prerequisites for bridging	Knowledge [10]	Specific areas of knowledge that are required to develop policy or support service delivery/enhancement for individuals aging with disability.
	Frameworks, models, theories [6]	Specific models and/or theories influencing beliefs regarding aging and/or disability, change processes, and/or service delivery/policy needs.
	Funding models [20]	Financial support that mandates or supports work involving aging and disability knowledge / teams / or targets issues and outcomes relevant to both groups or enables program delivery or evaluation.
	Policy [12]	Policy which mandates closer collaboration / joint planning across aging and disability.
	Supportive culture [17]	Culture of an organization (including attitudes, beliefs, values, actions) which in this case are supportive of innovation, collaboration, and change.
Bridging tasks	Liaison [3]	Communication or cooperation that facilitates a close working relationship between people or organizations.
	Research and evaluation [17]	Activities designed to produce new knowledge or make judgements about the merit, or significance of a subject, or to provide an assessment of need or of a problem to be addressed.
	Monitoring/ surveillance [7]	Ongoing, systematic collection, analysis and interpretation of health-related data to support the planning, implementation, and evaluation of public health practice.
	Training [8]	Teaching, enhancing or developing skills through context specific practice.
	Mentoring [3]	Relationship in which a more experienced or more knowledgeable person helps to guide a less experienced or less knowledgeable person.
	Education [13]	Providing structured information in a manner conducive to improving knowledge about matters related to health and/or functioning and/or service systems.
	Adapting services, or systems, to make services accessible to individuals with disability [23]	Adapting service model and/or removing barriers that prevent people accessing services, e.g. cultural, socioeconomic, physical barriers.
	Establishing health (related) services [15]	Providing health services and/or infrastructure where they were previously absent or inadequate to meet a specified population health need.
	Advocacy [20]	Mediating or arguing in the interest or on behalf of a person or group or population in relation to a particular matter.
	Advising [5]	Recommending a course of action in relation to changing or maintaining function, environments or behaviour.
	Partnerships/collaboration (82)^a^	Working together and cooperating with the person/client, health providers, and other relevant stakeholders.
	Planning [13]	Planning future interventions and/or working with stakeholders to develop their goals and identify strategies for achieving those goals.
	Awareness raising [7]	Promoting messages on a health or related topic, on areas of overlap between aging and disability, unique or specific issues, and of gaps in knowledge, policy, practice.
	Policy change [4]	Developing new or modifying existing policies (could be at an organizational level or broader e.g., provincial or federal)
	Policy development [11]	A process that involves specifying core concepts, desired policy outcomes and current knowledge on the issues to be addressed.
	Care coordination [29]	Navigating and facilitating the access, management and cohesion of services and supports for the client.
	Facilitation [37]^a^	Making the process easier, identify gaps, anticipate problems, help remove or negotiate barriers, and promote safe and effective connections to services and appropriate use of resources.
	Individualized client planning [10]	Working with the person to develop an individual proposal including setting client goals and priorities, and identifying actions, responsibilities and supports needed (services and resources) to achieve the goals.
	Assessment [14]	Evaluating the client’s health condition, functioning, environment, behaviour, situation or need for intervention.
	Navigation [10]	Finding the most appropriate pathway through systems, services, resources and supports for the client given their context.
	Referral [10]	The action of sending someone to see another person or place for consultation, review or further action, help or advice.
	Training and skills development [39]^a^	Teaching, enhancing or developing skills through context-specific practice, to clients or others in their circle of care.

^a^most frequently discussed bridging tasks

### Bridging tasks organized temporally and by level of care

After revisiting the extracted data and applying these concepts, a matrix was developed to organize the information and describe which bridging tasks occurred in different domains (policy, research and training and service delivery) and at different levels of care, as well as the relationships between bridging tasks, the inputs needed to undertake this bridging task, and the resulting outcomes. Table 3 presents the matrix developed for each of three domains: 1) policy; 2) research and training; and 3) health care and social service delivery. Information was separated as the nature of the bridging tasks, stakeholders involved, and desired outcomes, differed by domain.

**Table 3:** Specific examples of bridging within each domain applied to a matrix adapted from Thorncroft and Tansella (1998)

Domain	System level	Input	Tasks	Outcome
Policy	Macro	**• Policy development / changes–** that mandate joint collaboration and planning / degradation of structures promoting silos	**• Service delivery -** funding of services **• Collaboration and joint planning** & **service development**	**•** Not specified
Research and training	Macro	**• Policies** calling for collaboration and consideration of both aging and disability issues **•** Aging and disability research and training consortiums / professional associations / special interest groups **•** Funding	**• Planning,** **• Collaboration,** in **research** **• Advocacy and awareness raising**	**•** Knowledge creation through research and policy studies on aging and disability **•** Informed stakeholders on aging/disability issues
	Meso	**•** University centres in aging and developmental disability **•** Inter-agency **partnerships** / memorandums of agreement **•** Resources to support research (e.g., databases, data collection materials, protected staff time)	**• Monitoring / surveillance** of community needs / opportunities/needs for bridging **• Training & Education** **• Collaboration and joint planning** & **service development**	**•** Information on community needs to inform policy & service planning **•** Training to interdisciplinary teams / health workforce and research trainees **•** Curriculum development to train aging and disability workers
	Micro	**•** Supportive organizational culture **• Partnership** / **collaboration** between disability service and geriatric assessment clinic	**• Collaboration and joint planning** & **service development** **• Training and Mentoring** – rotating health professionals, providing exposure to different systems (disability or aged care specialty services)	**•** Not specified
Health and social service delivery	Macro	**• Policies** mandating collaboration, and attention to aging and disability issues **•** Inter-agency **partnerships** / memorandums of agreement **•** Shared values, theories and frames of reference	**• Collaboration, planning, advocacy, establishing and adapting services**	**•** Improving accessibility of care and the quality of services provided **•** Enhanced efficiency of service systems **•** Improving quality of life, inclusion of persons with disabilities, and aging in place.
	Meso	**•** Consultants / experts in aging and disability and agencies with dedicated staffing to provide information and facilitate access to resources **•** Funding **•** Inter-agency **partnerships** / memorandums of agreement **• Planning** Committees **•** Organizational **policies**	**• Collaboration, planning, establishing and adapting services** **• Advising, Liaising and Facilitating** – sharing of resources, enhanced collaboration across agencies, and advising on existing services/supports **• Training** of health workforce	**•** Improving the quality of care **•** Earlier and more accurate diagnosis of health issues **•** Improving quality of life, inclusion of persons with disabilities, and aging in place.
	Micro	**•** Social worker, nurses, case manager **•** Individuals living with disability, Curriculum / training initiatives to develop community skills in bridging	**• Training and skill development –**for clients and families **• Establishing services, Care coordination, Assessment, Liaising, Facilitating, Navigation** **• Mentoring**	**•** Facilitating timely and appropriate access to care, system navigation, aging in place, enhanced client choice regarding care **•** Inclusion of adults with IDD in community groups for older adults
	Nano	**•** Social worker, nurses, case manager	**• Individualised client planning, Care coordination, Navigation, Facilitating, Referral**	**•** Not specified

*Note- bridging tasks identified in our review are bolded to distinguish them from inputs and outcomes. Bridging may be a task in one domain and an input in another. Some tasks were observed to facilitate bridging in other areas*

In the domain of policy, bridging activities were described at the macro level, specifically policy development, or policy changes, and collaboration/partnerships. The primary stakeholder group discussed was the Administration for Community Living in the United States, responsible for interpreting and developing policies pertaining to services for older adults and individuals with disability [55, 57]. The legislation most frequently discussed to facilitate bridging was the Older Americans Act in the United States and specifically the different amendments to the act at the end of the twentieth century, which authorized state funding for older adults with disability services, and required greater collaboration between agencies serving older adults and those supporting individuals with disability [46]. No studies evaluated outcomes of the bridging activities in the policy domain, however, policy implementation was noted to drive other bridging tasks in the health care and social services domain including, collaboration and planning across aging and disability services to support service development with the goal of enabling older adults and those with disabilities to fully participate in their communities [24]. Although policy changes were noted to have had success in bringing together departments involved in aging with those working with disability, some authors also noted the absence of policy frameworks to address key issues affecting those aging with disability (e.g., accommodation/housing needs or retirement issues) [26, 28, 45] and the ongoing need for more attention to policy development involving those with expertise in aging and disability.

In the domain of research and training, bridging activities were described at the macro, meso and micro levels. At the macro level, bridging activities were related to funded research programs, and either national- or international-level planning and awareness raising through major events/conferences discussing intersecting and overlapping issues related to aging and disability [31, 45]. At the meso level, bridging involved planning and collaboration, research related to local consumer needs to inform planning activities, as well as education of health professionals [34, 37]. Conversely, at the micro level, bridging related to more specific training and mentoring of health professionals, so that those working in disability field could enhance their knowledge of aging and vice versa [32]. Stakeholders included researchers, health professionals and individuals aging with disability and their caregivers who participated in research and planning activities. Outcomes of bridging activities were related to knowledge creation that could inform policy and services to enhance the inclusion and quality of life of individuals aging with disability, and enhancing the quality of service delivery by establishing a more knowledgeable and skilled workforce.

In the domain of health care and social service delivery bridging occurred at all levels (macro or country; meso or community; micro or team/organization; and nano or patient level). Bridging tasks at all levels included planning, and collaboration between individuals or organizations with a focus on aging/aged care and those serving individuals with disability, adapting services and systems to meet the needs of all individuals, and establishing new programs or services [29, 41, 50]. Advocacy was a bridging task observed primarily at a macro level to raise awareness of policy needs and system level challenges in service delivery for individuals aging with disability [59, 60]. Education and training for health professionals was primarily observed at a meso level to insert specific expertise on aging or disability into community level services [65]. Care coordination tasks were observed at the micro and nano levels [32, 35]; stakeholders involved in these bridging tasks were often described in general terms (e.g., state and area agencies on aging/disability, planning committee, consultant with expertise in aging and disability). One specific group involved in bridging at the meso level were Aging and Disability Resource Centres established within the United States of America to assist individuals with disability and older adults to access services and supports [8]. At the micro level, certain professions were described as being suited to enact bridging, including social workers [54] and nurses [36]. Aging in place [26], improving access to or the quality of care [29, 50], improving inclusion or participation of individuals with disability [25, 27, 30], and service coordination [35] were outcomes mentioned, or suggested implicitly when describing the bridging tasks. Importantly some authors noted that many of the bridging tasks occurring in health or social service delivery were time limited, small scale projects led by individuals or teams (thus occurring at micro or meso levels) [46, 47], and this was attributed to the lack of an overarching policy framework regarding aging with disability.

## Discussion

This scoping review synthesized the literature discussing bridging for individuals aging with disability, and characterized the contexts where bridging has occurred, the nature of the bridging tasks discussed in this literature, the stakeholders involved, and the intended outcomes of bridging. From 49 included studies, published between 1988 and 2017, 22 main concepts describing bridging tasks were identified and grouped into domains: policy (e.g., policy changes mandating collaboration between services in aged care and disability), research and training (e.g., monitoring community needs, and educating professionals working in aged care on disability issues and vice versa), and health and social service delivery (e.g., care coordination, adapting services/systems so they are accessible to individuals with disability).

Trends showed the number of published papers discussing bridging increased over time. This may reflect a growing interest in bridging, or in aging with disability (the body of literature we searched), or a rising number of scientific publications annually [66]. Historically, studies on aging have had a more limited representation of individuals living with disability, which may explain why most studies were published after 1990 [67]. It is also interesting to note that two thirds of the studies were published after 1999 (i.e., after the Olmstead decision), which may have spurred a greater discussion of bridging in scientific literature. Additionally, seminal research papers on bridging, for example, the Graz Declaration on Disability and Ageing published in 2006 [68], the Barcelona Declaration in 2009 [69], and the Toronto Declaration on Bridging Knowledge, Policy, and Practices in Aging and Disability published in 2012 [4], may have contributed to a broader discussion of bridging in scientific literature, particularly in relation to aging and disability. While outside of the scope of the present review, it would be useful for future work to investigate the impact that these bridging papers had on subsequent bridging in practice and on bridging research. Interestingly, when the perspectives in which bridging tasks have been discussed were evaluated, there were no trends in certain perspectives being increasingly apparent in certain decades (e,g., bridging being called for in earlier papers and bridging tasks being evaluated in more recent papers). This highlights the present and persistent need for bridging tasks despite the continuous discussion surrounding its importance over the past 30 years.

Another significant finding was the large proportion of studies discussing bridging focused on individuals aging with I/DD. The area of intellectual disabilities has pioneered research in areas related to bridging such as, case management, and care coordination between social, employment, housing and health services [17, 70]. This emerged in the 1960’s as part of the deinstitutionalisation process of severe mental illness (including I/DD) and the early development of integrated care in this field. The rationale for bridging made in the literature related to two themes 1) the aging population and 2) the fact that individuals with disability are living longer [1]. The latter trend has been discussed extensively in relation to individuals with I/DD, which may in part explain why a large proportion of bridging literature focuses on this group [6, 71].

Studies included in this review used a multitude of different terms to describe the population of study. Some papers used diagnostic labels to classify the population, whereas others used concepts (like aging with disability). This posed a challenge in conducting this review due to the terminology variation. ‘Disability’ as a concept is difficult to define, and therefore to search; and diagnostic terms often take on different meanings depending on the context of the studies [72]. However, based on this review it appears that most literature on aging with disability either uses a range of health conditions expected to have a long-term influence on individuals’ lives, or focuses specifically on individuals with I/DD. There were a large number of articles initially retrieved (~ 12,000) relative to those included, which is common in scoping reviews which have broader questions, and systematically search large bodies of literature [73]. The high number of papers initially retrieved supports the use of scoping review methods (versus a systematic review), and highlights a need to find research terms related to broad concepts like aging, disability, and bridging, that are both more specific and unambiguous.

Framing in research is important, as it can clarify the concepts and definitions important to a topic, and therefore shape how ‘bridging’ is understood [9]. There were 22 bridging tasks that emerged through this review, far more than initially described in the Toronto Declaration. The Toronto Declaration was intended to draw attention to the need for bridging and in doing so outlined some initial bridging tasks that emerged through discussions with experts in the area of aging with disability [3]. By examining discussions of bridging in published literature preceding and following the declaration it was expected that through this scoping review additional bridging tasks were identified. Tasks related to bridging in the Toronto Declaration were coordination, assessment, empowerment, policy, management, service delivery, and financing [4], and many of the 22 bridging tasks identified in the current review, relate to these concepts. For instance, navigation, individualized client planning, and care coordination align with the broad category of coordination tasks, and advocacy and advising fall within the broad category of empowerment tasks. Thus, the current review confirms and expands upon the bridging framework initially proposed in the Toronto Declaration. Additionally, several bridging tasks related to case management, a complex intervention designed to facilitate access to services and supports, and improve the inclusion of individuals with long-term health conditions [17]. Existing taxonomies to specify case management interventions may be useful to draw on when implementing and evaluating bridging particularly at micro or nano levels of care [17].

Because there were many bridging tasks described in our dataset (see Additional file 1: Appendix A), it was more meaningful to organise them according to the domain, and level of care. Interestingly more bridging examples were noted in relation to health care and social service delivery than policy or research and training. Mapping also enabled separation of the tasks themselves, from inputs driving these actions, and the outcomes that result. The matrices published in this paper could potentially be used to a) generate research questions (e.g., evaluating the effects of specific bridging tasks in different domains); b) inform the development of a taxonomy (a classification scheme guiding communication on this topic); or c) assist in the specification of bridging in research and practice so that these tasks can be replicated in other implementation or research initiatives [9, 12]. In the present review, it was found that most bridging tasks evaluated were on a micro level within health and social service delivery, and further descriptive research identifying the efficacy, scalability, and sustainability of such tasks would be beneficial. In addition, there were many countries with a range of health and social systems undertaking bridging. Further understanding this context along with the levels of government responsible for overseeing these systems would assist in our understanding of bridging tasks at a macro level and areas for policy development to support more sustainable bridging in practice.

Another purpose of this review was to identify any gaps in the bridging literature. Despite several studies focusing on individuals aging with I/DD, the bridging activities discussed were highly variable and this makes synthesizing the literature to evaluate the effects of bridging challenging at this stage. Furthermore, much of the literature was made up of qualitative studies discussing individuals’ experiences/perceptions of aging with disability, or commentaries calling for bridging. A next step for the field would be to move towards evaluating bridging activities, and perhaps a starting point may be to evaluate bridging tasks conducted with individuals with I/DD. Our findings suggest there is clearly a need for bridging within the I/DD population, and that more research is needed to evaluate these bridging tasks and how they influence individual client- and system-level outcomes. In addition, it will be important to understand the knowledge, skills, and attributes that professionals need to engage in bridging, and what training is needed for professionals (e.g., social workers, intellectual disability nurses, occupational therapists or other rehabilitation professionals) who may be enacting bridging in practice [36].

In addition to noting the general need for more research that evaluates bridging activities, it is also important to better understand the outcomes of bridging. In the current review the inputs and outcomes of bridging were less clearly described, and greater emphasis was on the task itself. Many outcomes were described at a high level (e.g., aging-in-place, quality of life), and were often quite distant to the actual bridging task. For example, joint planning committees may have important process outputs that need to be tracked in addition to higher level outcomes such as influencing aging-in-place and inclusion in society. Once outcomes have been more explicitly defined, it will allow for the evaluation of bridging tasks and further advancement of health service delivery for individuals aging with disability.

There are some limitations to this scoping review. Firstly, we chose to focus the search on studies discussing the literature on aging with disability, and restricted “disability” to the areas of developmental and neurological disability [12]. This was initially done as it provided more selective definitions of disability and was an area that already had a body of literature surrounding bridging. However, this definition does not capture studies discussing disability experiences related to other health conditions (e.g., cardiovascular disease or other chronic conditions). This, in addition to the fact that the search only included studies up until 2017, means that it is possible some studies on bridging aging with disability may not have been included, and the findings may not be generalizable to other populations. In addition, grey literature and studies published in languages other than English were not included, meaning examples of bridging discussed outside of the scientific literature or in other languages may have been missed. The latter may bias the findings towards specific health system issues, population trends, and bridging tasks that pertains to English speaking countries and health systems. Additionally, the review could have been strengthened by explicitly considering cross-country variations in health and social service systems and whether that influenced the bridging tasks discussed. This would be a useful direction for future research.

## Conclusions

In conclusion, this scoping review characterized a range of tasks designed to bridge aging and disability policies, health and social care services, and knowledge (through research and training). The matrix constructed depicts bridging processes at different levels of care, and provide an important foundation for future research on bridging aging and disability.

## Supplementary information

**Additional file 1.** Appendix A. Summary of included articles, and the bridging activities discussed. Descriptive information about the articles such as the type of article, the main objectives, and the bridging activities discussed.

## Data Availability

The datasets used and/or analysed during the current study are available from the corresponding author on reasonable request.

## Abbreviations

AMED: Allied and complementary medicine
CINAHL: Cumulative index to nursing and allied health literature
FICCDAT: Festival of international conferences on caregiving, disability, aging and technology
GOWD: Growing older with a disability
I/DD: Intellectual and/or developmental disability
