# Supplementary material for: A scoping review to characterize bridging tasks in the literature on aging with disability
Source: BMC Health Serv Res. 2020 Mar 4;20:170. doi: 10.1186/s12913-020-5046-5 (PMC7057667; doi:10.1186/s12913-020-5046-5)
Supplement: Supplementary file 1 — Additional file 1. Appendix A. Summary of included articles, and the bridging activities discussed. Descriptive information about the articles such as the type of article, the main objectives, and the bridging activities discussed. [file 12913_2020_5046_MOESM1_ESM.docx]

| Appendix A. Summary of included articles, and the bridging activities discussed | | | | |
| --- | --- | --- | --- | --- |
| **Citation + location** | **Study population** | **Type of article** | **Main objectives** | **Bridging activities discussed** |
| Ansello, 1988 (USA)^20^ | General/aging with disability | Commentary, policy analysis, discussion and/or theoretical paper | None specified | (1) The signing of the Developmental Disability Act Amendments of 1987; (2) Federal level collaboration between Administrations on Aging and Developmental Disabilities; (3) 1987 University of Maryland Center on Aging sponsored conference of 40 state directors of aging and DD |
| Bigby, 1997 (AUS)^21^ | Intellectual disability | Original research (qualitative) | Explore aging experiences of older individuals with ID who have spent their earlier life years living at home with their parents | (1) Facilitating aging in place in disability residential services; (2) Consultation and training can equip generic staff in aged care services on ways to engage those with ID; (3) Improving accessibility of services |
| Bigby, 1998 (AUS)^22^ | Intellectual disability | Original research (qualitative) | Explore patterns and experiences of service use among aging people with ID in Victoria Australia | (1) The collaboration of aged and disability services; (2) Policy to improve care for those aging with disability |
| Bigby, 2002 (AUS)^23^ | Intellectual disability | Commentary, policy analysis, discussion and/or theoretical paper | Analyse services offered to people aging with ID from each sector to provide future directions for policy and service development to ensure the needs of this population are met | (1) Complementing aged care services with specialist disability inputs and bridging gaps between aging and disability with specialist service; (2) The visibility of people with disability within aged-care systems; (3) Partnerships and joint planning to remove barriers; (4) Mandated joint planning between sectors; (5) Research and teaching consortiums on aging and disability |
| Bigby, 2008 (AUS)^25^ | Intellectual disability | Commentary, policy analysis, discussion and/or theoretical paper | Review policy to support the provision of similar opportunities to age in place for people with ID, and the reasons for its slow development. | (1) Australia’s disability policy; (2) Aging in place for individuals with ID; (3) Cross-sector pilot program established by the Department of Health and Aging as part of their Innovative Pool Disability Aged Care Pilot Initiative; (4) Cross-sector planning and partnerships; (5) Enquiry into Access and Equity in Aged Care recommendations |
| Bigby, 2010 (AUS)^26^ | Intellectual disability/ developmental disability | Commentary, policy analysis, discussion and/or theoretical paper | Compare development and implementation of accommodation support policies for people aging with ID in five liberal welfare states | (1) Policies, programs, declarations etc. aimed at (a) improving social participation, (b) enabling funding of specialist services and mainstream aging services, (c) training on aging issues |
| Bigby, 2005 (AUS)^24^ | Intellectual disability | Original research (qualitative) | Compare different models of day-support programs used by older people with ID | (1) Models of day programs for older people with disability; (2) Community inclusion and support from social networks included in new policy directions |
| Bigby et al., 2014 (AUS)^27^ | General/aging with disability | Original research (case study/program evaluation) | Explain the components of the transition to retirement program and the policy, funding, and service delivery environment | (1) Trained mentorship program for retirees with ID; (2) Coordination of individuals into mainstream groups |
| Borson, 2010 (USA)^28^ | General/aging with disability | Commentary, policy analysis, discussion and/or theoretical paper | Create framework for thinking about how cognitive changes, aging and disability may interact to help explain individual differences in coping, and to promote the inclusion of cognition in a comprehensive approach to assessment and care | (1) Future collaboration on developing assessment of cognitive decline in those aging with disability |
| Chng, Stancliffe, Wilson, & Anderson, 2013 (AUS)^29^ | Intellectual disability | Original research (quantitative) | Evaluate whether training mentors in active mentoring would yield increased engagement in community group activities by older adults with IDD | (1) Participation of individuals with ID who have retired within the mainstream community group through active mentorship |
| Clark, 2007 (CDN)^30^ | General/aging with disability | Commentary, policy analysis, discussion and/or theoretical paper | Examine literature on home care policy (from federal government, national organizations, and aging and disability communities) to find the deep meaning behind the home care policy discussion | (1) Collaboration between the aging and disability communities due to potential for a common values dimension to their unified narrative frame; (2) 1987 Wingspread Conference on Aging and lifelong Disabilities; (3) The Aging with Lifelong Disabilities Movement |
| Coogle, Ansello, Wood, & Cotter, 1995 (USA)^31^ | Developmental disability | Original research (mixed or multiple methods) | None specified | (1) Improvement of service coordination through community level projects; (2) Cross-training of personnel in both aging and DD service networks; (3) Joint planning activities and resource sharing among DD and aging networks |
| Cotten & Spirrison, 1988 (USA)^32^ | Developmental disability | Original research (case study/program evaluation) | None specified | (1) Staff training across networks to accept older people with MR and increase level of communication between agencies; (2) Liaisons linking aging and MR services; |
| Coyle, Putman, Kramer, & Mutchler, 2016 (USA)^33^ | Intellectual disability/ developmental disability | Original research (qualitative) | Assess the activities of aging and disability resource centers as they seek to serve older adults with ID and their family caregivers | (1) Aging and Disability Resource Center partnerships across aging and IDD service systems; (2) Cross-training of staff in regards to physical, cognitive and social changes in older adults with disabilities |
| Dawson et al., 2014 (AUS)^34^ | Intellectual disability | Original research (case study/program evaluation) | Further understand the lived experience of residents and employees of care facilities, to shed insight on how to facilitate processes of change across two organizations with different funding arrangements and policy initiatives, and to illustrate how stories capture sensemaking processes and act as transitioning devices that give sense to future change opportunities | (1) Collaboration between workers from disabilities and aged care services with research to identify and re-characterize problems in storying new pathways for tackling transition needs in those aging with ID |
| Doody, Markey, & Doody, 2013 (IRL)^36^ | Intellectual disability | Commentary, policy analysis, discussion and/or theoretical paper | Present the context of ID in Ireland and aging for people with ID in the context of increasing longevity and service provision | (1) Personnel which bridge aging and ID such as Registered Nurses in Intellectual Disability; (2) Health reforms in the Government of Ireland |
| Doody et al., 2012 (IRL)^35^ | Intellectual disability | Commentary, policy analysis, discussion and/or theoretical paper | Outline how disability nurse trained in ID can address the needs and concerns of people aging with ID and advocate for them as well | (1) Coordination of services through Registered Nurses in Intellectual Disability; (2) Development and adaptation of comprehensive gerontological assessment to includes seniors with ID; (3) Collaboration between workers specialized in ID and aged and primary health care workers |
| Fahey-McCarthy, McCarron, Connaire, & McCallion, 2009 (IRL)^37^ | IDD or DS with Dementia or Alzheimer’s disease | Original research (mixed or multiple methods) | Understand care concerns with the unique issues of supporting persons with ID affected by dementia, report on education and training needs of staff supporting persons with ID and advanced dementia, and the development and testing of an educational intervention designed to address these needs | (1) Collaboration between multidisciplinary team of members drawn from ID and specialist care services |
| Friedman et al., 2012 (USA)^38^ | Intellectual disability | Review/knowledge synthesis | Better understand the unique barriers to end of life care for aging adults with ID with an emphasis on hospice services | (1) Liaison to provide comprehensive end of life care of individuals; (2) Collaboration between palliative care specialists and professional working with people with ID; (3) Development of palliative care curriculum and educational program for staff caring for people with ID in long term care; (4) Coordination and collaboration between inpatient and community setting |
| Gibson, Rabkin, & Munson, 1992 (USA)^39^ | Developmental disability | Original research (qualitative) | Identify what key service providers in aging services network and developmental service network see as critical issues in providing services | (1) Cross-training of workers within the field of DD and aging; (2) Development of university level courses surrounding fields of service with older individuals with DD as clients |
| Hartley et al., 2015 (USA)^40^ | I/DD or DS with Dementia or Alzheimer’s disease | Commentary, policy analysis, discussion and/or theoretical paper | None specified | (1) Collaboration between Alzheimer's Association in Partnerships with the Linda Crinic Institute for Down Syndrome and the Global Down Syndrome Foundation for a workshop of AD and DS experts; (2) Establishment of Professional Interest Area for DS-AD to advance Alzheimer’s Research and Treatments of the Alzheimer’s Association; (3) Grant program sponsored by the associations to support work moving forward |
| Hassiotis, Strydom, Allen, & Walker, 2003 (UK)^41^ | I/DD or DS with Dementia or Alzheimer’s disease | Original research (case study/program evaluation) | Describe prospectively cognitive decline in people with ID due to DS or other causes | (1) Collaboration between Psychiatry for the Elderly and Intellectual Disabilities in West Essex to improve referral and screening; (2) Development of dementia questionnaire for those with I/DD; (3) Exposure to performing cognitive assessments in individuals with I/DD to enhance training |
| Hawkins & Eklund, 1989 (USA)^42^ | Developmental disability | Commentary, policy analysis, discussion and/or theoretical paper | Discuss direct planning outcomes and indirect process outcomes related to increased interaction among state-agency employees involved in the Indiana task force on aging and aged persons with DD | (1) The Indiana Project for planning and policy development for individuals aging with DD; (2) Interagency planning task force on aging/aged persons with DD |
| Hegland, 1991 (USA)^43^ | Mental retardation | Commentary, policy analysis, discussion and/or theoretical paper | None specified | (1) Training of MR/DD surveyors on age-elated issues; (2) Providing personal care in a community setting in residence houses for older clients with MR; (3) Growing Older Accessible Life Services to provide senior-centered activation/socialization for those with MR |
| Hobson et al., 2012 (UK)^44^ | I/DD or DS with Dementia or Alzheimer’s disease | Original research (case study/program evaluation) | Describe service improvement project to identify and screen all adults with DS over 30 years and establish function baselines and to set up database to facilitate early diagnosis of dementia | (1) Partnerships between Intellectual Disability and older adult services to aid diagnosis of individuals with I/DD and dementia; (2) Proactive screening project to identify/screen those with I/DD and create a database to facilitate early identification of dementia |
| Iezzoni, 2014 (USA)^45^ | General/aging with disability | Commentary, policy analysis, discussion and/or theoretical paper | Summarize major policy challenges addressed in 4 conference papers and mentioned during discussions among conference speakers and participants | (1) Bridging of historical divides between aging and disability fields; (2) Joint meeting with sponsors from the aging and disability fields |
| Janicki, McCallion, Force, Bishop, & Lepore, 1998 (USA)^46^ | General/aging with disability | Original research (mixed or multiple methods) | Explore the feasibility for area agencies on aging to incorporate within their day-to-day activities low-cost and low-tech outreach, linkages with DD agencies, and supports for households with an adult who has DD | (1) 1987 amendments to the Older Americans Act; (2) Collaboration between the New York State Office of Mental Retardation and Developmental Disabilities, the New York State Office for Aging, the New York Developmental Disabilities Planning Council and several university-based research and service centers; (3) Carer contact data form to document the demographics and needs of the households identified as well as the process used to establish contact |
| Keys & Factor, 2001 (USA)^47^ | Developmental disability | Original research (case study/program evaluation) | Identify and illustrate four core issues (leadership, member recruitment, collaborative chemistry, sustaining activity) of building empowering community coalitions | (1) Coalition building between aging and developmental disability field; (2) Joint initiatives such as grant proposal to ensure inclusion of older people disabilities in the community; (3) Discussion with agency leaders around issues related to aging and DD and about their awareness and interest with the issues |
| Kropf, Malone, & Welke, 1993 (USA)^48^ | Developmental disability | Commentary, policy analysis, discussion and/or theoretical paper | Describe an educational model used to prepare students to work with older people who have MR | (1) Graduate level course on aging and MR through Leadership Training Program in Aging and DD at the University of Georgia; (2) Collaboration between Georgia University Affiliated Program and the University of Georgia Gerontology Center in developing the program |
| McCallion & Janicki, 1997 (USA)^49^ | Developmental disability | Original research (quantitative) | Determine to what extent AAAs target families caring for older adults with DD and to establish the level of organizational preparedness among AAAs target families caring for older adults with DD | (1) Collaboration between the New York State Office of Mental Retardation and DD, The New York State Office for Aging, the New York Developmental disabilities planning council and several University based research and services centers; (2) Amendments to the Older Americans Act including provision that required older persons with disabilities to be more readily services within services provided by the aging network; (3) AAA service delivery - estimating number of aging families with a member with a DD in the area, ensuring people with DD service in existing aging programs such as senior centers and nutrition sites; (4) Agreement between local aging and DD agencies to work together, and have regular and formal communication between the service systems |
| McCarron et al., 2003 (IRL)^50^ | I/DD or DS with Dementia or Alzheimer’s disease | Commentary, policy analysis, discussion and/or theoretical paper | Draw upon data available on the population with ID and dementia, consider the policy context and argue for a specific model of service provision | (1) Model created by geriatric psychiatry and generic memory clinics for service development and care provision for dementia in ID; (2) Development of mobile dementia care clinics incorporating expertise from specialties such as old age psychiatry, the up-skilling of specialist Intellectual Disability Nurses and other members of the multidisciplinary ID team; (3) American Association on Mental Retardation Guidelines for Clinical Assessment and Management of dementia developed by Alzheimer's Association |
| McCarron, McCallion, Fahey-McCarthy, Connaire, & Dunn-Lane, 2010 (IRL)^51^ | Intellectual disability | Original research (qualitative) | Understand staff perceptions of critical issues in caring for persons with ID and advanced dementia and to develop interventions to address cross training concerns | (1) Development of collaborative training program between ID services and specialist palliative care provider; (2) Dementia and memory clinics aimed to identify individuals with ID and symptoms and train staff; (3) Post Graduate Diploma in Dementia and Intellectual Disabilities by the School of Nursing and Midwifery, Trinity College Dublin |
| McDaid, Cieza, & Gomez, 2009 (ESP)^52^ | General/aging with disability | Commentary, policy analysis, discussion and/or theoretical paper | Provide brief snapshot of the key themes and ideas emerging during 3 days of presentation and debate on methods, policy and practice | (1) Person-centered approach to bridging: focus on active aging and restoring people to full health rather than avoiding illness or managing disability and in regards to age-friendly cities; (2) USA legislation created system of social support services for older people and those with ID; (3) Members of the research, policy and practice communities in the fields of aging and disability came together in Barcelona for the first international conference on bridging knowledge in long-term care and support |
| McGinley, 2016 (USA)^53^ | Intellectual disability/ developmental disability | Review / knowledge synthesis | Explore utilizing a constructionist perspective, how challenges around aging and disability evolved from a nonissue to an impending crisis. | (1) Toronto Declaration/Growing Older with Disability Conference; (2) American Association on Intellectual and Developmental Disabilities End-of-Life Care for Children and Adults with IDD and position statement; (3) New York State Office of People with Developmental Disabilities' Aging Information Corner |
| Parish & Lutwick, 2005 (USA)^54^ | Developmental disability | Commentary, policy analysis, discussion and/or theoretical paper | Describe how social workers unique position can be used to provide optimal services for individuals aging with DD | (1) Facilitating linkages with agencies engaged in providing long-term care services to the population with DD through social workers; (2) Coalition building between people with DD and elderly people |
| Parkinson & Howard, 1996 (USA)^55^ | Developmental disability | Commentary, policy analysis, discussion and/or theoretical paper | Not specified | (1) Administration on Aging and Administration on Developmental Disabilities signed memorandum of understanding calling upon aging and disability agencies to collaborate; (2) Development of the Council of Aging Developmental Disabilities of Greater New York; (3) Task Force on Aging and Developmental Disabilities consisting of co-chairs from aging and disability fields; (4) Cross-training by task |
| Putnam, 2014 (USA)^6^ | General/aging with disability | Commentary, policy analysis, discussion and/or theoretical paper | Describe how to build capacity and support to support the aging with disability population using structural bridges in the aging and disability fields as a part of rebalancing initiatives | (1) Structural bridging across aging and disability Long Term Services and Supports through Aging and Disability Resource Centers; (2) NIH student support on aging with disability through investigator-initiated research programs; (3) Collection of data from individuals aging with disability to determine unmet needs and identify best practices in older individuals and translate these interventions to those aging with disability |
| Robinson, Dauenhauer, Bishop, & Baxter, 2012 (USA)^56^ | Intellectual disability/ developmental disability | Commentary, policy analysis, discussion and/or theoretical paper | Bring attention to the limited presence of social workers in the IDD field, the lack of educational opportunities to support the current and future social workers in this field, and the impact of these trends on health disparities experienced by people who are aging with IDD | (1) Program in Aging and DD (PADD) at the University of Rochester Strong Center for DD; (2) Partnerships between PADD and Lakes Geriatric Education Center to improve education and training of geriatricians and health care practitioners; (3) Planning and leadership of end of life care for individuals with DD can be supported by social workers |
| Ronneberg, Peters-Beumer, Marks, & Factor, 2015 (USA)^57^ | Intellectual disability/ developmental disability | Original research (mixed or multiple methods) | Understand current awareness and practice within Adult Day Services regarding end of life care; and how palliative care providers understand/perceive adult day services; determine if collaborations exist between these organizations; barriers to collaboration and future directions for research | (1) Partnership between Administration on Aging with the Administration on Intellectual and Developmental Disabilities and the Department of Health and Human Services on Disability to form the Administration for Community Living; (2) Topic-specific training around needs of IDD population for end-of-life providers; (3) Targets for collaborative activities between IDD and aging services; (4) Communication and care planning across organization |
| Salvador-Carulla, Putnam, Bigby, &; Heller, 2012 (ESP)^3^ | General/aging with disability | Commentary, policy analysis, discussion and/or theoretical paper | Provide an illustrative research agenda mentioning fields that may be explored, approaches that can be followed and provides key references to guide read in quest | (1) Bridging aging and disability theory through the International Classification of Functioning, Disability and Health |
| Seltzer, Krauss, Litchfield, & Modlish, 1989 (USA)^58^ | Mental retardation | Original research (quantitative) | Systematically analyze the extent to which elderly persons with MR were served by aging network agencies in one state and to identify salient organizational factors associated with such integration activities | (1) Hiring of staff trained in MR in aging networking programs; (2) Interagency collaboration between aging networks and MR service system |
| Service & Hahn, 2003 (USA)^59^ | Intellectual disability/ developmental disability | Commentary, policy analysis, discussion and/or theoretical paper | Review historic and current information about aging people with DD and discuss the role of nurses in the support and care of these individuals | (1) The International Association on the Scientific Study of Intellectual Disabilities Fact Sheet on Aging; (2) Interagency collaboration catalyzed by nurses in I/DD and aging; (3) Curriculum development for inclusion of I/DD in aging and aging in I/DD for health care professionals, staff and families |
| Sheets, Wray, & Torres-Gil, 1993 (USA)^61^ | General/aging with disability | Commentary, policy analysis, discussion and/or theoretical paper | Propose a broader conceptualization of geriatric rehabilitation that encompasses rehabilitation interventions that support independent living | (1) Creating of the Research and Training Center on Aging and Disability; (2) Aging, Disability and Rehabilitation Task Force fostered coalition among rehabilitation, health care, and aging professionals and providers; (3) Geriatric Rehabilitation Policy Alternatives Project; (4) The 1985 National Conference on Disability and Aging |
| Sheets & Liebig, 2005 (USA)^60^ | General/aging with disability | Commentary, policy analysis, discussion and/or theoretical paper | Describe changing demographics of disability and the needs for supportive living environments; identify issues that may hinder or buttress efforts to build collations between the aging network and disability community; illustrate recent public policies in the US that serve both populations | (1) Coalitions to enhance leverage of aging interest groups; (2) Aging and Disability Resource Centers assisting states in creating single integrated systems for individuals aging with disabilities; (3) Fair Housing Act Amendments (1998), Americans Disability Act, Assistive Technology Act, Olmstead decision and New Freedom Initiative; (4) White Conference on Aging Mini-conference on Aging and Disability |
| Sutherland Smith, Thyer, Clements, & Kropf, 1997 (USA)^62^ | Developmental disability | Original research (case study / program evaluation) | Report on an evaluation of coalition building forums around the state of Georgia, which brought together aging and disability service providers; discuss outcome of forum, such as whether providers implemented information and techniques from these forums to reduce fragmentation of service options for this population | (1) University Affiliated Program for Persons with DD at the University of Georgia; (2) Forums attended by consumers and families, aging and DD personnel, administrators from both networks and allied professionals; (3) Funding for collaboration between medical school and assistive technology program, participation in a health fair with other agencies, planning a conference on disabilities, and arranging for volunteer experiences for people with DD at senior center; (4) Coalition building forum across 2 years bringing together DD and aging service, consisted of training components on networking, collaboration, and education between providers of different service networks |
| Tedrick, 1990 (USA)^63^ | Developmental disability | Commentary, policy analysis, discussion and/or theoretical paper | Explore the problems seen in defining DD when the concept of aging is added; discuss difficulties in assessing how many of these adults exist; review recent policy initiatives at the federal and state level | (1) University Affiliated Program for Persons with DD who are aging; (2) Bridging knowledge through a call for proposals by the National Institute on Aging on the social, psychological and biological aspects of aging; (3) Wingspread Conference in June 1987 brought together an elite group of specialized in MR/DD and aging |
| Wark et al., 2013 (AUS)^64^ | Intellectual disability | Original research (qualitative) | Examine issues surrounding aging with an ID within rural areas and identify possible areas of change to policy and practice | (1) Development of a joint training agenda comprised of aged and disability networks |
| Washko, Campbell, & Tilly, 2012 (USA)^65^ | General/aging with disability | Commentary, policy analysis, discussion and/or theoretical paper | Address 3 interrelated issues critical to the fields of aging and disability, and to the well-being of the unique population of individuals aging with disabilities | (1) Aging and Disability Resource Centers; (2) Community Living Initiative which sought to identify and promote ways to improve access to housing, community supports and independent living arrangements for individuals with disabilities and older adults; (3) Funding for cross-disability research and training centers on aging with a disability; (4) Partnerships between the aging and disability communities |
| Webber, Bowers, & McKenzie-Green, 2010 (AUS)^66^ | Intellectual disability | Original research (qualitative) | Explore how group home staff caring for aging residents with ID responded to the development of age- related health changes of their residents, and to identify some of the reasons that people with ID are relocated to residential aged care | (1) Improving availability of resources and promoting aging in place; (2) Collaboration between health care professionals within the current setting or outside agencies to provide holistic care of medical conditions; (3) Education through consultants to inform staff and agency managers; (4) Collaborating with experts in aged care services to discuss accessibility and availability of resources |

ID, intellectual disability; IDD, intellectual and development disability; DD, developmental disability; MR, mental retardation; DS, Down syndrome; AD, Alzheimer’s disease; AAA, area agencies on aging; NIH, National Institutes of Health;
